# Simulation Study for Designing a Dedicated Cardiac TOF-PET System

**DOI:** 10.3390/s20051311

**Published:** 2020-02-28

**Authors:** Sandra Oliver, Laura Moliner, Víctor Ilisie, J.M. Benlloch, M.J. Rodríguez-Álvarez

**Affiliations:** Instituto de Instrumentación Para Imagen Molecular (i3M) Centro mixto CSIC-Universitat Politècnica de València, Camí de Vera s/n, 46022 Valencia, Spain; lmoliner@i3m.upv.es (L.M.); vicil@upvnet.upv.es (V.I.);

**Keywords:** positron emission tomography (PET), PET imaging, dedicated cardiac system

## Abstract

The development of dedicated positron emission tomography scanners is an active area of research, especially aiming at the improvement of lesion detection and in support of cancer treatment and management. Recently, dedicated Positron Emission Tomography (PET) systems with different configurations for specific organs have been developed for improving detection effectiveness. Open geometries are always subject to distortion and artifacts in the reconstructed images. Therefore, the aim of this work is to determine the optimal geometry for a novel cardiac PET system that will be developed by our team, and determine the time resolution needed to achieve reasonable image quality for the chosen geometry. The proposed geometries consist of 36 modules. These modules are arranged in two sets of two plates, each one with different configurations. We performed Monte Carlo simulations with different TOF resolutions, in order to test the image quality improvement in each case. Our results show, as expected, that increasing TOF resolution reduces distortion and artifact effects. We can conclude that a TOF resolution of the order of 200 ps is needed to reduce the artifacts, to acceptable levels, generated in the simulated cardiac-PET open geometries.

## 1. Introduction

Each year, cardiovascular diseases cause 3.9 million deaths in Europe, accounting for 45% of all deaths [1]. Functional information can be obtained with Positron Emission Tomography (PET) systems, an imaging modality widely used in cancer detection, which is also relevant in diagnosis and prognosis of heart diseases [2,3,4]. This modality is able to provide information on heart perfusion and the functionality on the myocardium tissue. Estimation of myocardial perfusion contains independent prognostic information about future major cardiac events, [5,6]. Moreover, perfusion assessment is also useful in the monitoring of the effectiveness-of-risk reduction strategies [7]. In view of the previous results, dedicated cardiac PET devices are currently being developed [8,9].

The main advantages of designing dedicated PET systems [10,11], compared with whole body PETs (WBPET) are the reduction of costs and dimensions and, moreover, they often present an increased sensitivity. The sensitivity is strongly dependent on the scanner design [10,12]. However, in our particular case, as the area of highest sensitivity must be the heart region, the detectors are placed closer to it, and, therefore, even if the global sensitivity of the system might be lower, it will be optimized for the cardiac region that is of interest in this study. WBPET systems have cylindrical geometries, with diameter about 70 cm of gantry (on average, bore diameter), while dedicated PET systems show smaller dimensions or even open geometries, in order to improve the access to the explored organ. These open geometries exhibit a lack of angular information, introducing artifacts and distortion effects in the final reconstructed images. Time of flight (TOF) information provided by the detectors plays an important role in the reduction of these non-desired effects for open PET geometries.

PET detection is based on the concept of Line Of Response (LOR). When two annihilation photons simultaneously impact two different detectors within a predetermined time-window (coincidence window), we consider that an event has occurred along this LOR. If TOF information is not available, the annihilation process is equiprobable all along the LOR, as there is no time information corresponding to the gamma ray impact. When introducing TOF information, it allows for the assignment of a Gaussian probability for a region of the LOR that contains the annihilation point. The width of the Gaussian distribution, and thus the spatial uncertainty of the annihilation point along the LOR, is directly proportional to the time resolution of the detection system. TOF information provides a better estimation of the position of the annihilation process along the LOR compensating, in part, the lack of angular information produced by the open geometries, reducing artifacts in the reconstructed images, and improving the image quality [13,14].

Currently, commercial TOF dedicated electronics reach time resolutions in the range of 200–400 ps [15,16] even though experimental systems have reached up to 150 ps with only two detector blocks [17,18,19].

In this context, our study aims to determine the optimal geometry for a dedicated open cardiac PET device considering different TOF resolutions. We simulated different open geometries and compared them with a full ring design. As a consequence of this study, a prototype device will be developed according to the optimal configuration determined in terms of geometrical design and achievable TOF resolution.

## 2. Materials and Methods

### 2.1. Geometry System Description

**Figure 1:**
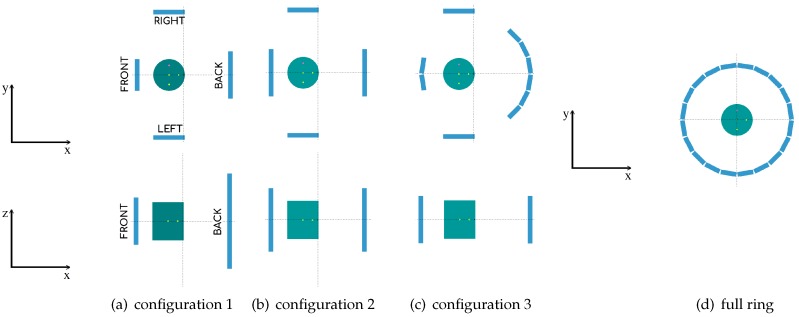
2D images for the proposed PET cardiac geometries of two sets of two parallel plates with a phantom as a heart. For configurations 1 (**a**), 2 (**b**), and 3 (**c**), we show the XY view (top) and the XZ view (bottom). For full ring configuration (**d**) we show XY view.

The different geometries corresponding to the proposed system contain 36 detector modules. (This number of detectors was mainly chosen due to budget limitations. As mentioned previously, the current project is meant to be cost-competitive, and so the maximum cost estimation for the development of this prototype is about 220,000 euros, which puts an upper limit on the total number of detectors.) Each detector module consists of 50 mm × 50 mm and 15 mm high density (7.1 g/cm${}^{3}$) LYSO scintillator blocks. The corresponding geometry configurations are shown in Figure 1a–c and Table 1 contains their specifications. These proposed geometries were specifically designed to obtain a high angular coverage at the heart region, maintaining the number of detectors constant. Additionally, in order to make our study more robust, we compare our results to the ones obtained with a full ring PET of 60 modules, as shown in Figure 1d. (The aim is to determine which geometry could be the one that obtains the best results compared to a closed ring of similar diameter, as closed rings will always outperform open geometries. Therefore, this particular choice (of the radius) is, by far, the most conservative one, as a ring with larger radius would give poorer results.)

For the open geometries, we consider that the frontal plate is located close to the chest wall and the back plate is located against the patient’s back. With this configuration, the two parallel plates can be placed in close proximity to the heart, covering a high angular sample of the organ in exploration. The geometry is asymmetric maximizing the sensitivity of the system at the heart location, placed at about 2/3 of the front-back distance. For configurations 1 and 2, the front-back plates are separated 28 cm and the left-right plates 38 cm (see Figure 1a,b). For configuration 3, the ring diameter is of 32.6 cm and the left-right plates are separated 38 cm. The full ring PET scanner has 32.6 cm of diameter (see Figure 1c,d).

### 2.2. Monte Carlo Simulations

For our study, we performed Monte Carlo (MC) simulations with a hand-made subroutine of PENELOPE (version 2014) [20] based on the GATE PET coincidence sorter [21]. PENELOPE is a general purpose code system for MC simulations of coupled electron, positron and photon transport in matter [22,23], which accurately models of particle interactions in an arbitrary material for energies between 50 eV and 1 GeV. A comprehensive comparison of simulation results with experimental data are available in the literature [24]. Therefore, for the energy range of interest in this work, it has demonstrated reliability of both the adopted interaction models and the tracking algorithm. For this study, we simulate positron sources and restrict possible coincidences only for the plates that are placed directly opposite to each other. The output of these simulations is a list-mode data set.

#### 2.2.1. Scanner Simulations with Different TOF Values

**Figure 2:**
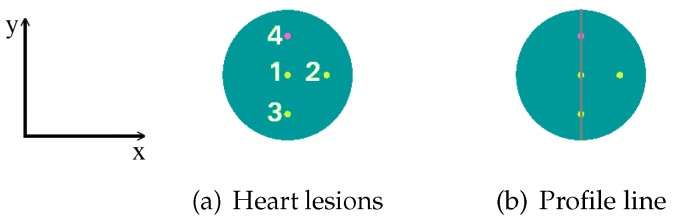
Spheres that represent heart lesions, numbered from 1 to 4. The hot spheres 1, 2 and 3 are located at (x,y) coordinates given by (0,0), (3,0) and (0,−3) cm, respectively, with respect to the center of the cylinder. The cold sphere is located at (0,3) cm from the center of the cylinder. The three hot spheres present a specific volume activity ratio 8:1, with respect to the background (**a**); the colored line that defines the image profiles is presented later on (**b**).

The simulation of the scanners uses a phantom placed at an estimated heart location. This phantom is a cylinder of 10 cm of diameter and 12 cm height, containing four spheres of 5 mm of diameter each. As we can see in Figure 2a, the three hot spheres with 8:1 activity concentration ratio (in reference to the background activity of 25 $\frac{kBq}{mL}$) are located at (x,y) coordinates given by (0,0) cm for sphere number 1, (3,0) cm for sphere number 2, and (0,−3) cm for sphere number 3, with respect to the center of the cylinder. The cold sphere, number 4, (with no activity) is located at (0,3) cm from the center of the cylinder. Later on, in Section 3.2.1, as in some cases we will not be able to solve the 5 mm spheres, we shall use 8 mm diameter spheres instead, with the same coordinates. Moreover, we add profiles of the reconstructed images for the spheres with these two diameters along the *y*-axis of the phantom. The lines that correspond to these profiles are shown in Figure 2b.

In this analysis, each sphere represents a heart lesion. In real cardiac lesions, inflammation, blood flow obstruction, death of muscle tissue, and others occur, so that the metabolism, and therefore the glucose uptake, is altered. The 4-sphere phantom does not pretend to be, at this stage of the analysis, a representative model of possible injuries; however, it is used as a measure of the image contrast, as it is commonly performed in NEMA protocols.

For the analysis of the obtained results, the criteria that we have adopted for saying that a lesion (sphere) can be solved or not is the following: we define the capability to detect a hot (or cold) lesion in the reconstructed image based on the signal intensity profile of the concerned feature. Hot (respectively cold) lesions are considered as detected if their intensity profiles were visibly distinguishable above (or below for cold features) the background signal level.

The simulated acquisition time is 100 s, using ${}^{18}F$ considering a continuum positron energy spectrum in water with the maximum end-point energy of 0.634 MeV as implemented in the PENELOPE code. The considered scintillators for the simulations are LYSO crystals (7.1 g/cm${}^{3}$). The simulations are performed with 600, 400 and 200 ps of full width at half maximum (FWHM) time resolution, and without TOF information, for each considered geometry. The obtained list-mode data are reconstructed with TOF-OSEM [25,26], using Jacobs projector [27] based on Siddon’s algorithm [28], without attenuation and scatter corrections. In order to include TOF information in the reconstruction, Jacobs ray tracing algorithm is modified weighting the forward and backward projections of the data with a Gaussian kernel centered in the estimated annihilation point along each LOR [29]. The image reconstructions are performed corresponding to the considered TOF resolutions, and without TOF information. The voxel size of the reconstructed images is set to 1.4 mm × 1.4 mm × 1.4 mm, and the Field Of View (FOV) contains 200 × 200 × 200 voxels. Therefore, the FOV dimensions are 28 cm × 28 cm × 28 cm for all geometries, including the full ring scanner. The iterations and subiterations used in the reconstruction algorithm are 9 and 2, respectively, for the non-TOF case, 7 and 2 for the reconstruction with 600 ps, 6 and 2 for 400 ps and 5 and 2 for 200 ps. The choice of the applied number of iterations tries to reach a good compromise between sufficient contrast noise ratio (CNR) and restricting noise. Note that, with increasing TOF resolution, a lesser number of iterations was needed to obtain a good image convergence.

#### 2.2.2. Simulations for Sensitivity Evaluation

The sensitivity of a PET scanner assesses its ability to detect positron annihilation gamma rays, and consists in a measure of the rate at which coincidence events are detected. The system’s sensitivity is defined as the number of events measured, compared to the total number of events that correspond to the source activity in events/sec or Bq. The sensitivity is measured according to NEMA NU-2012 [30] for the considered geometries. In order to obtain the sensitivity value, we simulated a ${}^{18}F$ capillary tube of 700 mm length, 3.9 mm of diameter and an activity of 10 MBq, inserted in a set of five concentric Aluminium sleeves. As NEMA-NU 2012 suggests, the inner diameters of the sleeves range in between 3.9 mm and 16.6 mm and the outer diameters between 6.4 mm and 19.1 mm. The capillary tube is located trans-axially at the center of the FOV. With these configurations, we performed the MC simulations and reconstructed the images with a voxel size of 0.8 mm × 0.8 mm × 0.8 mm. Afterwards, the total counts for each sleeve were obtained and extrapolated in order to find the counts without the metal attenuation. The total sensitivity of the scanner is finally obtained using the following equation:

(1) $$S_{tot}\left(cps/kBq\right)=\frac{R_{0}}{Activity}\;$$

where $R_{0}$ is the extrapolated value for the number of counts per second with no attenuation.

#### 2.2.3. Simulations for Spatial Resolution Evaluation

**Figure 3:**
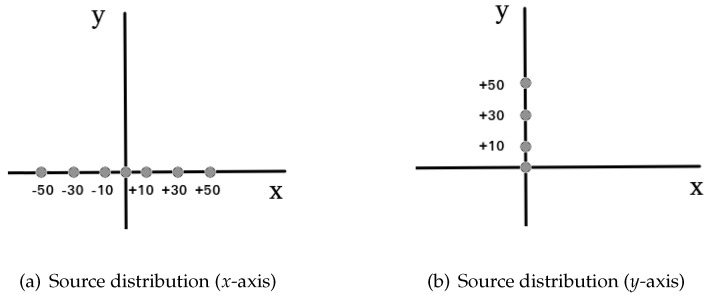
Capillary sources distribution along the *x*-axis (**a**), and *y*-axis (**b**) for the spatial resolution calculation.

The spatial resolution of a system determines its ability to differentiate two adjacent details in the reconstructed image. With this measurement, we characterize the widths of the Point Spread Function (PSF) of the radioactive sources within the reconstructed image. The width of the PSF is defined by the FWHM and the full width at tenth maximum (FWTM) of the reconstructed image of the sources along the three spatial directions: radial, tangential, and axial. The spatial resolution is calculated for the different geometries and compared with the full ring geometry, according to NEMA NU-2012. For this purpose, we simulated a capillary source of 3MBq${}^{18}F$ with 1 mm of diameter and 1 mm length. The capillary source was moved across the transverse FOV ($FOV_{T}$) at seven radial positions along the *x*-axis (radial direction), namely the center of the $FOV_{T_{radial}}$, ± 10 mm, ± 30 mm, and ± 50 mm from the center of the $FOV_{T_{radial}}$, as shown in Figure 3a. NEMA-NU 2012 protocol recommends placing the capillary tube at 10 mm and 100 mm. However, for our configurations, the 100 mm distance lies outside the detection area of the left-right plates. For the open geometries, we consider as the center of the FOV, the point where the center of the plates coincide. Due to the asymmetry of these geometries with respect to the *x*-axis, we moved the source both sides from the center of the $FOV_{T_{radial}}$ along the *x*-axis (negative and positive). For the *y*-axis (tangential direction), the source was moved at four positions, namely the center of the $FOV_{T_{tang}}$, +10 mm, + 30 mm and + 50 mm from the center of the $FOV_{Ttang}$. This was done along the positive side only because all of the analyzed geometries are symmetric with respect to this axis, as shown in Figure 3b. The measurements were repeated at $\frac{3}{8}$ of the axial FOV ($FOV_{A}$), again, as NEMA-NU 2012 indicates. For the full ring geometry, we calculated the spatial resolution at radial positive positions (due to its symmetry) and the same was done for the $\frac{3}{8}$ measurements.

We performed the MC simulations for the above-mentioned configurations for a 100 s acquisition. The images were reconstructed with a voxel size of 0.8 mm × 0.8 mm × 0.8 mm and with five iterations and two subiterations. The volumetric FWHM and FWTM for the source in each position were also calculated. The volumetric FWHM is defined as the product of the FWHM of each axis: $FWHM_{x}\times FWHM_{y}\times FWHM_{z}$, and the same is valid for the volumetric FWTM.

### 2.3. Image Quality Indicators

In this section, we define four metrics to compare the simulated (ideal/ground truth) image with the reconstructed images of the phantom for each geometry. These metrics are the root mean square error (RMSE), the peak signal-to-noise ratio (PSNR), the normalized root mean square distance (NRMSD), and the normalized mean absolute distance (NMAD) [31], defined as follows:

(2) $$RMSE=\sqrt{\frac{1}{N}\sum_{n=1}^{N}{\left(u\left(n\right)-u_{true}\left(n\right)\right)}^{2}}\;$$

(3) $$PSNR=10\cdot log_{10}\left(\frac{MAX^{2}\left(u_{true}\right)}{\frac{1}{N}\sum_{n=1}^{N}{\left(u\left(n\right)-u_{true}\left(n\right)\right)}^{2}}\right)\;$$

(4) $$NRMSD=\sqrt{\frac{\sum_{n=1}^{N}{\left(u\left(n\right)-u_{true}\left(n\right)\right)}^{2}}{\sum_{n=1}^{N}{\left({\bar{u}}_{true}-u_{true}\left(n\right)\right)}^{2}}}\;$$

(5) $$NMAD=\frac{\sum_{n=1}^{N}\left|u\left(n\right)-u_{true}\left(n\right)\right|}{\sum_{n=1}^{N}\left|u_{true}\left(n\right)\right|}\;$$

where *u* denotes the reconstructed image, $u_{true}$ denotes ideal image, $MAX(u_{true})$ denotes the maximum value of the intensity of the ideal image, |x| denotes the absolute value of *x*, and ${\bar{u}}_{true}$ denotes the average intensity in the volume of interest (VOI). The VOI is selected as the set of voxels occupied by the ideal image. The index of the voxels within the VOI is denoted by *n*, and *N* is the total number of voxels.The total number of voxels was adapted for each geometry configuration in order to always cover the whole FOV).

These image quality indicators quantify voxel-to-voxel differences inside the VOI. When the reconstructed image and the ground truth image are equal, the RMSE value is zero, while the PSNR value tends to infinity. On the other hand, NRMSD is an estimation of uniformity. Its value is 1 when the reconstructed image is entirely uniform with the average intensity ${\bar{u}}_{true}$, which is the average of the ideal image. Finally, NMAD tends to 1 when the voxel intensity of the reconstructed image is negligible with respect to the ideal image, and tends to zero if both images are equal.

In order to determine the ability to detect the spheres inside the phantom, with a quantitative analysis, we used the contrast recovery coefficient (CRC). This indicator provides quantitative accuracy for the spheres. For this calculation, different VOIs are drawn over the hot and cold spheres. The selected VOIs are cylindrical VOIs with the same diameters as the spheres. The axial extension of the VOIs is two voxels. For each VOI, we obtain the mean counts: $C_{H}$ for the hot spheres and $C_{C}$ for the cold sphere. Due to distortions produced in the reconstructed images near the hot spheres, to determine the background counts, we choose an annular region, such as a toroid, with an inner radius of 5 mm for the center of the hot spheres, and outer radius of 10 mm for the spheres of 5 mm. For the case of the spheres of 8 mm, we choose an inner and outer radius of 6.5 mm and 11.5 mm, respectively. From the voxels inside the toroid, we estimated the mean background counts, $C_{B}$. The VOIs used for the background have the same thickness as the sphere VOIs. The CRC for the hot spheres is calculated as in Equation (6) using the NEMA-NU 2012 definition, where R is the true-to-hot-background activity concentration ratio that has a value of 8 in our simulations. For the cold sphere, CRC is estimated by the Equation (7):

(6) $$CRC_{H}=\frac{\frac{C_{H}}{C_{B}}-1}{R-1}\;$$

(7) $$CRC_{C}=1-\frac{C_{C}}{C_{B}}\;.$$

## 3. Results

### 3.1. Monte Carlo Simulation Results

#### 3.1.1. Reconstructed Images for Different TOF Value Simulations

**Figure 4:**
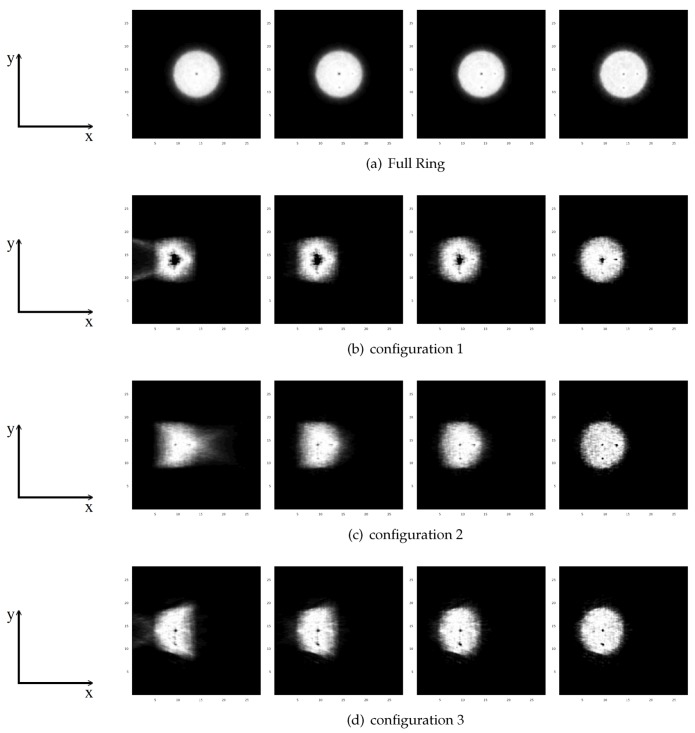
Central transverse slice for the reconstructed images for a 10-cm diameter cylindrical phantom, for a full ring (**a**), configuration 1 (**b**), configuration 2 (**c**), configuration 3 (**d**). For each set, the four images (from left to right) are: non-TOF, 600 ps, 400 ps, and 200 ps.

Figure 4 shows the central transverse slice for the specified cylinder phantom, with 10 cm of diameter, located at the estimated position of the heart and with three hot spheres inside. Moving from left to right, in this figure, we can observe the benefits of using TOF information for the reconstruction algorithm. Notice that the reconstructed images are not calibrated to quantitative units. These images are obtained with a forward and backward projection of the recollected data, i.e., detected events. The number of events is related with the activity and dose received in each voxel. Thus, regions with higher activity are shown with higher intensity (black, for hot spheres), and regions with less intensity indicate lesser deposited dose (gray/white color for the phantoms).

For the proposed open geometries, there are significant artifacts and distortions in the non-TOF reconstructed images due to the missing angular views, when compared with full ring non-TOF reconstructed images. Improving the TOF resolution, we obtain a higher quality image in which the artifacts are significantly reduced. We can also conclude that, in order to detect the three hot spheres, a 200 ps time resolution is needed. In cardiac imaging, structures are normally larger; however, higher image resolution will always provide more information about the damaged or swollen region. In this sense, if it is possible to detect slightly inflamed regions, injuries could be prevented in earlier stages of the development of the disease.

#### 3.1.2. Sensitivity Evaluation Results

With the complete study of the sensitivity for each geometry configuration, we obtain the values shown in Table 2. As expected, higher sensitivity values are obtained with the full ring scanner, followed by configurations 1 and 2 that have similar sensitivity values. Finally, design number 3 provided the lowest sensitivity.

#### 3.1.3. Spatial Resolution Evaluation Results

After the complete study of spatial resolution for each geometry, we obtain the values reported in Table 3 and Table 4 corresponding to the volumetric FWHM and FWTM obtained for each tested design. For all the proposed geometries, the spatial resolution improves at the center of the FOV. For configurations 1 and 3, we obtain better results with an offset of +10 mm when compared to the center of the FOV. This effect is due to the geometric asymmetry of the configuration with respect to the *x*-axis.

**Figure 5:**
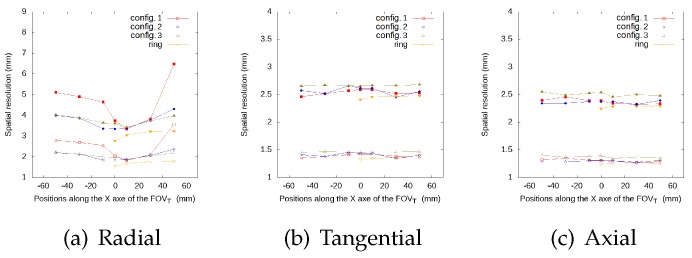
FWHM (empty symbols) and FWTM (solid symbols) values (in mm) for each geometry, for the source at different positions along the *x*-axis of the $FOV_{T}$, radial (**a**), tangential (**b**) and axial (**c**) values, at the center of the $FOV_{A}$.

**Figure 6:**
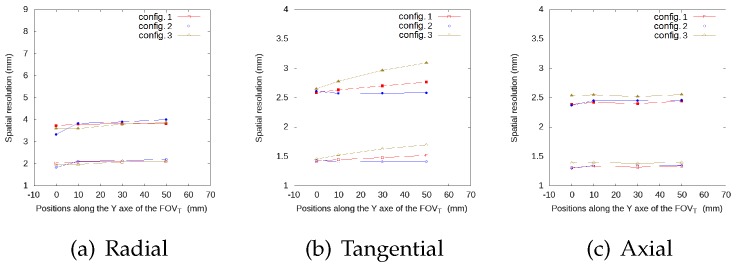
FWHM (empty symbols) and FWTM (solid symbols) values (in mm) for each geometry, for the source at different positions along the *y*-axis of the $FOV_{T}$, radial (**a**), tangential (**b**) and axial (**c**) values, at the center of the $FOV_{A}$.

**Figure 7:**
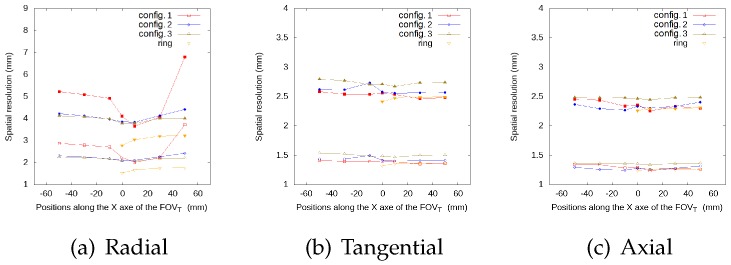
FWHM (empty symbols) and FWTM (solid symbols) values (in mm) for each geometry, for the source at different positions along the *x*-axis of the $FOV_{T}$, radial (**a**), tangential (**b**) and axial (**c**) values, at 3/8 from the center of the $FOV_{A}$.

**Figure 8:**
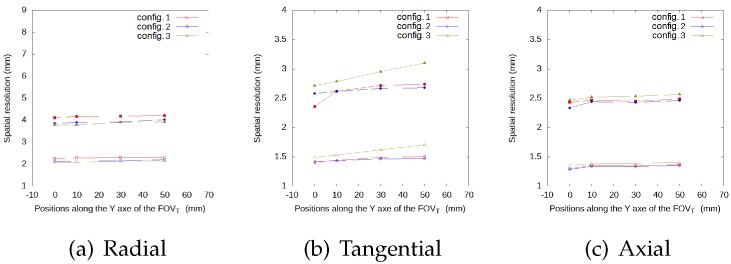
FWHM (empty symbols) and FWTM (solid symbols) values (in mm) for each geometry, for the source at different positions along the *y*-axis of the $FOV_{T}$, radial (**a**), tangential (**b**) and axial (**c**) values, at 3/8 of the center of the $FOV_{A}$.

Figure 5, Figure 6, Figure 7 and Figure 8 represent the radial, tangential, and axial values of the FWHM and FWTM for the different positions of the source at the center of the $FOV_{A}$ and at $\frac{3}{8}$ from the $FOV_{A}$, respectively. For the tangential and axial components, the FWHM and FWTM values for all geometries are similar, and also similar to the full ring geometry. In the radial direction, configuration 2 presents the best values for FWHM and FWTM, its spatial resolution is more homogeneous along the $FOV_{T}$, and its values are closer to the ring geometry, when compared to configurations 1 and 3.

### 3.2. Impact of TOF in Image Quality Indicators

**Figure 9:**
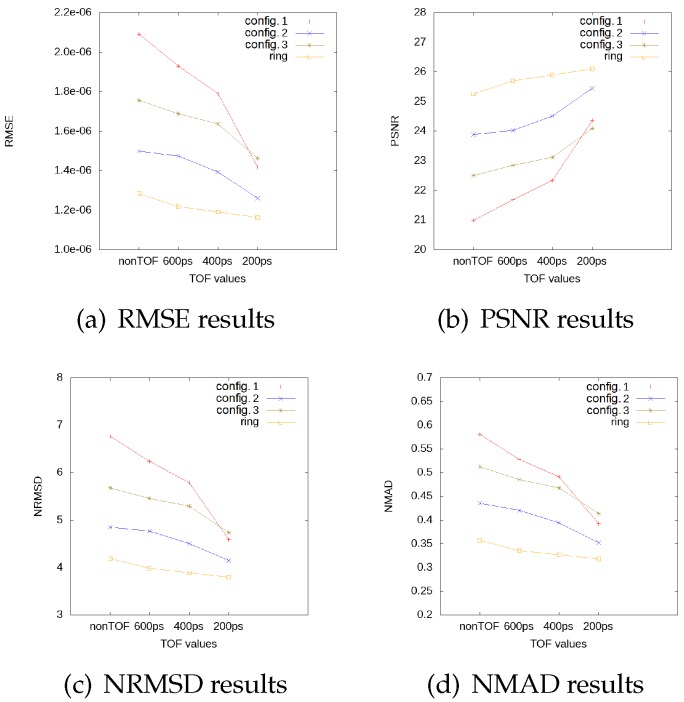
Image quality indicator values, RMSE (**a**), PSNR (**b**), NRMSD (**c**) and NMAD (**d**), for each geometry as functions of time resolution for the TOF scanner. In each plot, the results from a full ring scanner are also shown for comparison, for each TOF value.

In Figure 9, we can observe the image quality indicators for each geometry. The full ring geometry values for RMSE, NRMSD, and NMAD slightly decrease when we improve the time resolution, and the PSNR value slightly increases, which demonstrates that the ideal and the reconstructed images are more similar and present less differences when we consider better time resolution. For configuration 2 with a time resolution of 200 ps, the image quality indicators are similar to those obtained with the full ring configuration. The general behavior for all open geometries is a linear decrease for RMSE, NRMSD, and NMAD, and a linear increase for PSNR, with a soft slope for TOF values for the three considered cases: non-TOF, 600 ps, and 400 ps. Nonetheless, we observe a drop in these indicators for a time resolution of 200 ps. This TOF value seems to provide a reconstructed image which is almost artifact-free. Moreover, with this time resolution, we can detect the hot spheres for all geometries. In view of these results, we conclude that configuration 2 with a TOF value of 200 ps provides the best results, an almost artifact-free image, and the best values for the quality indicators. For this reason, we shall name this configuration from now on the optimal cardiac PET configuration.

The behavior of the configuration described by configuration 3 offers in general better results when compared to configuration 1. However, this is no longer true for a TOF value of 200 ps because configuration 1 presents better values for the image quality indicators. This is due to the fact that, for TOF values greater than 200 ps, the reconstructed images present artifacts and distortions, and one is not able to solve correctly the hot spheres. When the artifacts are reduced, for configuration 3, we can not detect lesion 2 due to a deformity that appears in the reconstructed image. This is mainly because of the module arrangement in the frontal plate.

#### 3.2.1. Contrast Recovery Coefficient Evaluation

As we can see in Figure 4, we are not able to detect the cold sphere clearly, despite having a time resolution of 200 ps and only the spheres with a larger diameter than 5 mm can be detected. In this sense, we performed three additional simulations with different diameters for hot and cold spheres using the different system configurations defined previously.

**Figure 10:**
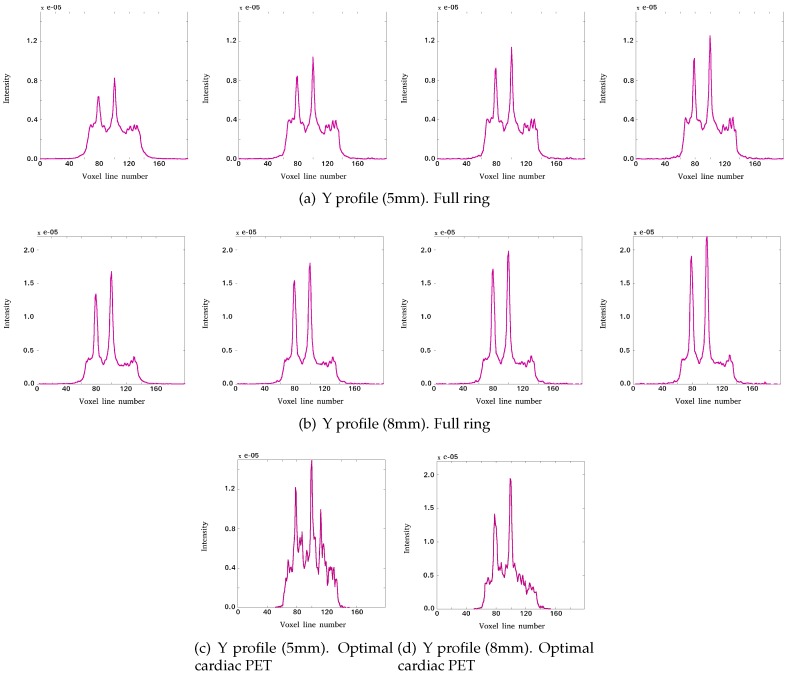
Y profiles of the reconstructed images showing lesions 3, 1, and 4 for: full ring with (left to right) non-TOF, 600 ps, 400 ps, and 200 ps TOF value, with spheres of 5 mm diameter (**a**) and 8 mm (**b**) diameter inside the phantom; optimal cardiac PET with 200 ps TOF value with spheres of 5 mm (**c**) and 8 mm (**d**) diameter inside the phantom. The profiles are presented as *Intensity* (in arbitrary units) versus number of line voxel.

In Figure 10, the count profiles are shown along the *y*-axis, and we can observe that, with diameters of 5 mm for the spheres inside the phantom, the cold sphere cannot be detected. However, it is possible to detect it with a phantom with spheres of 8 mm diameter.

**Figure 11:**
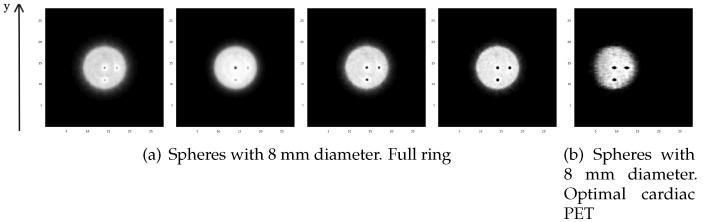
Central transverse slice for the reconstructed images of a phantom with three hot spheres and a cold sphere of 8 mm diameter for: (**a**) full ring with (left to right) non-TOF, 600 ps, 400 ps and 200 ps TOF value; (**b**) optimal cardiac PET with a 200 ps TOF value.

In Figure 11, we show the reconstructed images of the central transverse slice for a cylindrical phantom of 10 cm diameter with spheres of 8 mm of diameter, for the full ring geometry (a) and for the optimal cardiac PET geometry with 200 ps (b). We calculate the CRC for the hot and the cold spheres for each time resolution and for 5 mm and 8 mm diameters.

**Figure 12:**
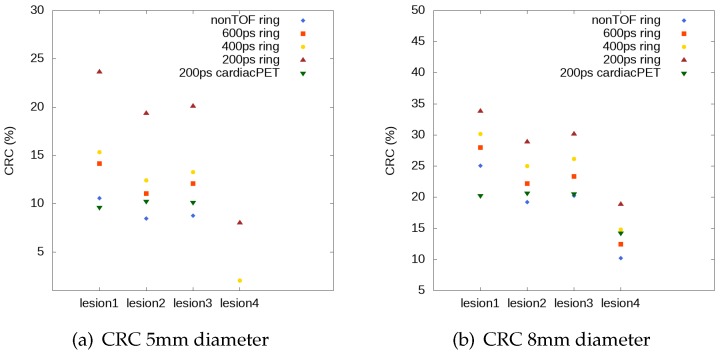
CRC values for the three hot spheres and the cold sphere (lesions 1, 2, 3, and lesion 4) with diameter of 5 mm (**a**) and 8 mm (**b**); results for full ring and optimal cardiac PET geometry.

Figure 12 shows the estimated CRC values for the three hot spheres and for the cold sphere (lesions 1, 2, 3, and lesion 4). The CRC values for lesion 4 are shown only for those TOF values for which this lesion can be solved, according to the results presented above. The CRC values improve in the case of optimal cardiac PET system with time resolution of 200 ps. In this case, the cold sphere can be detected and the results are closer to the full ring profiles, which correspond to 5 mm diameter spheres.

The results show that TOF imaging has an impact on the CRC values obtained for the full ring scanner. As we can see, for the spheres of 5 mm in diameter, we are able to detect the cold sphere only when TOF values are 400 ps or 200 ps. For larger values of the sphere diameters such as 8 mm, we can detect the cold sphere also, without TOF information, only for the full ring geometry. We therefore observe that the CRC values are better for larger diameter of the spheres.

## 4. Discussion and Conclusions

In this work, we presented the design optimization of an open-PET system used for cardiac research. For this study, three open geometries were proposed and analyzed, all of them formed by 36 modules arranged in different configurations. The study shows that, due to the reduced angular coverage of the tested open-PET configurations, a time resolution of 200 ps is required in order to detect the hot spheres of the simulated phantom. In view of these results, similar image quality indicators are obtained for the reconstructed images for configuration 2 with 200 ps, and for the full ring configuration without TOF information. All tested open PET configurations showed inferior performance, again, when compared to the full ring PET. Notice that, because there are no standards for dedicated systems, it is not possible to compare the results obtained with the NEMA protocol with other dedicated systems [32,33,34,35,36,37,38,39].

The image quality tests show that the image resolution for configuration 2 is the closest with respect to the full ring scanner configuration. Its spatial resolution is more homogeneous along the trans-axial FOV when compared to the values obtained for the other geometries. Therefore, the open PET design 2 is considered as a suitable candidate for future developments of an open configuration for cardiac PET research.

We investigated the contrast improvement due to TOF capability for the full ring geometry, for the reconstructed images without TOF information and with TOF values of 600 ps, 400 ps, and 200 ps, and for the optimal cardiac TOF-PET system with 200 ps TOF. We have studied the CRC values of hot and cold spheres. We thus conclude that the optimized open PET cardiac configuration is suitable for the study of cold and hot structures in the cardiac region that have at least a size of 8 mm. With the comparison between CRC values, the full ring geometry shows that there is a significant contrast improvement for a better timing resolution. The results for the optimal cardiac TOF-PET system show that the CRC values are similar to non-TOF values for full ring scanner for lesions 2, 3, and 4, when the spheres have a diameter of 8 mm. On the other hand, for lesion 1, the full ring geometry achieves a 20% higher CRC value for the non-TOF reconstruction when compared to the optimal cardiac TOF-PET system with TOF of 200 ps.

The sensitivity analysis provides similar results for configuration 1 and configuration 2. There is a negligible difference of about 2% between them, and it is mainly due to the fact that both configurations subtend approximately the same solid angle. Configuration 3 presents a worse sensitivity value because of the arrangement of the modules, which, in this case, presents a smaller subtended solid angle.

In conclusion, we have shown that the TOF information plays a key role in defining the design of an open PET system conceived for cardiac imaging. Improved TOF timing resolution reduced quantitative artifacts and image distortion. In view of the reported results, it is feasible to obtain artifact-free reconstructed images, using an open-geometry dedicated cardiac PET system i.e., configuration 2, with 200 ps TOF performance. With this TOF resolution, the obtained images are comparable to the ones obtained with a full ring PET scanner. This TOF resolution is experimentally achievable with current SiPM technology coupled to monolithic crystal blocks [40]. Thus, finally, a prototype device for a dedicated cardiac TOF-PET system corresponding to the geometry number 2 will be developed by our team, reaching a compromise between lower costs and reasonably acceptable image quality.

## Figures and Tables

**Table 1:** Geometry description. Modules organized as a matrix distribution for each plate of the open geometries.

Geometry	Matrix Modules	
	Front−Back	Left−Right
Configuration 1	2 × 3 − 3 × 6	2 × 3 − 2 × 3
Configuration 2	3 × 4 − 3 × 4	2 × 3 − 2 × 3
Configuration 3	2 × 3 − 6 × 3	2 × 3 − 2 × 3

**Table 2:** Total sensitivity values in cps/kBq for each geometry configuration.

Geometry	$S_{tot}$ (cps/kBq)
Configuration 1	3.87
Configuration 2	3.81
Configuration 3	2.17
Full ring	5.86

**Table 3:** Spatial resolution for each geometry. Volumetric FWHM ($mm^{3}$) and FWTM ($mm^{3}$) at different radial positions along the *x*-axis of the transverse FOV ($FOV_{T}$). The central values correspond to capillary tubes at the center of the $FOV_{A}$, and the 3/8 values correspond to an axial displacement of 3/8 from the center of the $FOV_{A}$.

Radial $FOV_{T}$		Configuration 1		Configuration 2		Configuration 3		Full Ring	
Offset (mm)		Center	3/8	Center	3/8	Center	3/8	Center	3/8
−50	FWHM	5.08	5.48	3.98	4.34	4.50	4.75	-	-
−50	FWTM	30.72	33.14	24.08	26.28	27.22	28.76	-	-
−30	FWHM	5.01	5.21	3.77	4.10	4.25	4.64	-	-
−30	FWTM	30.30	31.56	22.80	24.80	25.73	28.10	-	-
−10	FWHM	4.72	4.83	3.49	4.07	4.02	4.42	-	-
−10	FWTM	28.55	29.24	21.10	24.65	24.36	26.77	-	-
0	FWHM	3.82	4.10	3.43	3.84	4.01	4.18	2.55	2.59
0	FWTM	23.10	24.84	20.75	23.22	24.25	25.31	15.43	15.71
+10	FWHM	3.35	3.46	3.47	3.75	3.69	4.09	2.92	2.98
+10	FWTM	20.27	20.94	20.99	22.68	22.34	24.75	17.7	18.04
+30	FWHM	3.70	3.87	3.55	4.09	4.10	4.50	3.04	3.14
+30	FWTM	22.40	23.41	21.49	24.75	24.83	27.26	18.43	19.00
+50	FWHM	6.32	6.42	4.35	4.53	4.36	4.53	3.07	3.17
+50	FWTM	38.23	38.87	26.35	27.40	26.38	27.42	18.61	19.18

**Table 4:** Spatial resolution for each geometry. Volumetric FWHM ($mm^{3}$) and FWTM ($mm^{3}$) at different tangential positions along the *y*-axis of the transverse FOV ($FOV_{T}$). Center values correspond to capillary tubes at the center of the $FOV_{A}$, and the 3/8 values correspond to an axial displacement of 3/8 from the center of the $FOV_{A}$.

Tangential $FOV_{T}$		Configuration 1		Configuration 2		Configuration 3	
Offset (mm)		Center	3/8	Center	3/8	Center	3/8
0	FWHM	3.82	4.10	3.43	3.84	4.01	4.18
0	FWTM	23.10	24.84	20.75	23.22	24.25	25.31
+10	FWHM	4.02	4.45	4.01	4.10	4.22	4.38
+10	FWTM	24.35	26.92	24.30	24.84	25.57	26.53
+30	FWHM	4.09	4.60	4.09	4.20	4.71	4.81
+30	FWTM	24.78	27.82	24.78	25.42	28.54	29.13
+50	FWHM	4.29	4.74	4.22	4.38	5.07	5.15
+50	FWTM	25.94	28.70	25.55	26.52	30.66	31.17
